# Continuous renal replacement therapy in COVID-19—associated AKI: adding heparin to citrate to extend filter life—a retrospective cohort study

**DOI:** 10.1186/s13054-021-03729-9

**Published:** 2021-08-19

**Authors:** Eduardo de Oliveira Valle, Carla Paulina Sandoval Cabrera, Claudia Coimbra César de Albuquerque, Giovanio Vieira da Silva, Márcia Fernanda Arantes de Oliveira, Gabriel Teixeira Montezuma Sales, Igor Smolentzov, Bernardo Vergara Reichert, Lucia Andrade, Victor Faria Seabra, Paulo Ricardo Gessolo Lins, Camila Eleuterio Rodrigues

**Affiliations:** Hospital das Clínicas, University of São Paulo School of Medicine, Av. Dr. Arnaldo, 455, 3º andar, sala 3310, São Paulo, SP CEP 01246-903 Brazil

**Keywords:** Acute kidney injury, COVID-19, Continuous renal replacement therapy, Citrate, Heparin, Filter lifespan, D-Dimer

## Abstract

**Background:**

Coronavirus disease 2019 (COVID-19) may predispose patients to thrombotic events. The best anticoagulation strategy for continuous renal replacement therapy (CRRT) in such patients is still under debate. The purpose of this study was to evaluate the impact that different anticoagulation protocols have on filter clotting risk.

**Methods:**

This was a retrospective observational study comparing two different anticoagulation strategies (citrate only and citrate plus intravenous infusion of unfractionated heparin) in patients with acute kidney injury (AKI), associated or not with COVID-19 (COV + AKI and COV − AKI, respectively), who were submitted to CRRT. Filter clotting risks were compared among groups.

**Results:**

Between January 2019 and July 2020, 238 patients were evaluated: 188 in the COV + AKI group and 50 in the COV − AKI group. Filter clotting during the first filter use occurred in 111 patients (46.6%). Heparin use conferred protection against filter clotting (HR = 0.37, 95% CI 0.25–0.55), resulting in longer filter survival. Bleeding events and the need for blood transfusion were similar between the citrate only and citrate plus unfractionated heparin strategies. In-hospital mortality was higher among the COV + AKI patients than among the COV − AKI patients, although it was similar between the COV + AKI patients who received heparin and those who did not. Filter clotting was more common in patients with D-dimer levels above the median (5990 ng/ml). In the multivariate analysis, heparin was associated with a lower risk of filter clotting (HR = 0.28, 95% CI 0.18–0.43), whereas an elevated D-dimer level and high hemoglobin were found to be risk factors for circuit clotting. A diagnosis of COVID-19 was marginally associated with an increased risk of circuit clotting (HR = 2.15, 95% CI 0.99–4.68).

**Conclusions:**

In COV + AKI patients, adding systemic heparin to standard regional citrate anticoagulation may prolong CRRT filter patency by reducing clotting risk with a low risk of complications.

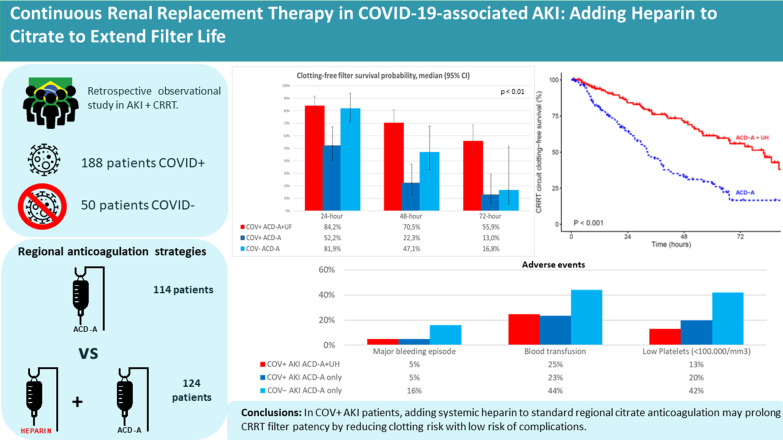

**Supplementary Information:**

The online version contains supplementary material available at 10.1186/s13054-021-03729-9.

## Introduction

Severe acute respiratory syndrome coronavirus 2 (SARS-CoV-2) is an extremely lethal agent that results in coronavirus disease 2019 (COVID-19), which has caused more than a million deaths worldwide [1]. In the intensive care unit (ICU), up to 30% of COVID-19 patients develop acute kidney injury (AKI) and consequently require renal replacement therapy (RRT) [2].

In patients with severe COVID-19, there have been reports of endothelial damage and subsequent thrombotic events, accompanied by elevated levels of fibrinogen and D-dimer, which are also predictors of a poor prognosis [3, 4]. There have also been reports of multiple peripheral and cerebral infarcts, as well as myocardial infarction with ST-segment elevation, and an increased incidence of pulmonary embolism [5–7]. Although some retrospective studies have suggested that anticoagulation with heparin is beneficial in patients with severe COVID-19 [8] that is still controversial and there is a need for more robust scientific evidence.

Hypercoagulability increases the risk of early clotting of the extracorporeal circuit in patients on continuous renal replacement therapy (CRRT). Some reports have suggested that, among critically ill patients on CRRT, the rates of premature filter change and dialysis downtime are higher in those with COVID-19 than in those without [9, 10].

The purpose of this study was to evaluate the impact that different anticoagulation strategies, namely regional citrate anticoagulation (RCA) only and RCA plus unfractionated heparin, have on the risk of CRRT circuit clotting.

## Methods

### Study design and population

This was a single-center, retrospective, observational study, involving critically ill patients treated at a large tertiary care hospital. From January 2019 to July 2020, all patients with AKI requiring CRRT were considered for inclusion in the study.

Until the end of 2019, before the COVID-19 pandemic, the standard of care for CRRT at our institution was continuous venovenous hemofiltration (CVVH) with prefilter dilution, although continuous venovenous hemodialysis (CVVHD) or continuous venovenous hemodiafiltration (CVVHDF) was used in some patients. In January 2020, the CRRT standard became either CVVHD or CVVHDF, because we believed that there might be a higher risk of filter clotting in CVVH [11]. All COVID-19 patients arrived at our institution in 2020, when the new standard of care was CVVHD or CVVHDF. To better compare COV + AKI and COV − AKI regarding filter clotting, only patients receiving CVVHD or CVVHDF were included in this study. The first CVVHD or CVVHDF procedure was included in the analysis, regardless of the number of readmissions.

We defined AKI on the basis of the Kidney Disease: Improving Global Outcomes (KDIGO) criteria [12]. We defined COV + AKI as AKI from any cause in SARS-CoV-2–positive patients, with diagnostic confirmation by real-time reverse transcriptase–polymerase chain reaction, or in patients who had symptoms of upper or lower respiratory tract infection and chest computed tomography findings suggestive of COVID-19. We defined COV − AKI as AKI from any cause in patients who did not have COVID-19.

### CRRT prescription

One of three machines was used for CRRT: Diapact (B. Braun Medical, Inc., Melsungen, Germany), with a 1.0–2.3 m^2^ polysulfone high-flux filter (Diacap HI; B. Braun Medical, Inc.), for CVVHD or CVVH; Prisma (Gambro, Lund, Sweden), with a 0.9 m^2^ membrane (AN69 M100 filter set; Gambro) for CVVHDF; or Multifiltrate (Fresenius, Bad Homburg vor der Höhe, Germany), with a 1.8 m^2^ membrane (AV1000 set; Fresenius), also for CVVHDF. Post-filter ionized calcium (iCa) was measured three times per day.

The decision to start CRRT was based on standard clinical guidelines. In all cases, bicarbonate-buffered solution was used. Filters were routinely changed after 72 h, or sooner if any dysfunction was detected. A prefilter pressure > 270 mmHg was considered indicative of filter clotting. The prescribed dialysis dose was 30 ml/kg of body weight/h. When CVVHDF was performed, the protocol was two-thirds dialysis and one third hemofiltration.

### Anticoagulation strategies

The main predictor of interest was the type of anticoagulation strategy employed. Before the COVID-19 pandemic, RCA for CRRT at our hospital was performed with anticoagulant citrate dextrose solution formula A (ACD-A; JP Indústria Farmacêutica, Ribeirão Preto, Brazil). Each 1000 ml of ACD-A contains 74.8 mmol trisodium citrate and 38.1 mmol citric acid (i.e., 112.9 mmol of citrate/L). The RCA was carried out with 3 mmol of ACD-A per liter of treated blood, with a target post-filter iCa concentration of 1.0–1.4 mg/dl. In April 2020, to counter the higher risk of RRT circuit clotting in COV + AKI patients, the standard RRT anticoagulation protocol was changed to include prefilter infusion of unfractionated heparin in all COV + AKI patients, unless heparin use was contraindicated or the patient was already receiving systemic heparin for another reason. In addition, the ACD-A dose was increased to 4 mmol/L, with a target post-filter iCa concentration of < 1.0 mg/dl. Unfractionated heparin was infused prefilter at a fixed rate of 10 U/kg of body weight/h, which was not increased to reach a target activated partial thromboplastin time (aPTT), although it was decreased if that value was greater than 2.0 times the control value or discontinued if the patient experienced any anticoagulation-related adverse event. For patients receiving systemic heparin for indications other than RRT anticoagulation, the decision to alter the dose of or discontinue heparin was made by the ICU physician. We divided the sample into three groups, by the diagnosis of COVID-19 and the anticoagulation strategy employed: COV − ACD-A only; COV + ACD-A only; and COV + ACD-A plus unfractionated heparin (COV + ACD-A + UH). The COV + ACD-A + UH group included patients receiving heparin via the protocol described for CRRT and those receiving systemic heparin for indications other than RRT anticoagulation.

### Data collection

At ICU admission, demographic and clinical data were recorded. Prior to CRRT, we collected physiological data, including vital signs, and biochemical data. In COVID-19 patients, we collected the D-dimer values that were determined closest to CRRT initiation and those that were determined closest to the clotting event. For the COV − AKI patients, D-dimer levels were not measured.

During the first 72 h of CRRT, clinical variables were evaluated, as were filter patency and any adverse events that could be related to the anticoagulation agent, such as bleeding (minor or major) and a low platelet count (< 100,000/mm^3^). A hemorrhagic event was defined as any bleeding event reported in the electronic medical record. It was defined as a major bleeding episode if accompanied by a drop in hemoglobin of ≥ 1 g/dl in 24 h or as a minor bleeding episode if the drop in hemoglobin was < 1 g/dl in 24 h. In COV + AKI patients, the serum D-dimer levels determined closest to CRRT initiation were used in order to stratify the patients.

We investigated CRRT-associated electrolyte disturbances from the initiation of CRRT until the day following the first filter replacement. They were defined as electrolyte disturbances not present at the initiation of CRRT and meeting the following criteria: hypokalemia (serum K < 3.5 mEq/L), hyperkalemia (serum K > 5.0 mEq/L), hypophosphatemia (serum P < 2.5 mg/dL), hyperphosphatemia (serum P > 6.0 mg/dL), hyponatremia (serum Na < 130 mEq/L), hypernatremia (serum Na > 150 mEq/L), hypocalcemia (serum ionized Ca < 4.3 mg/dL), hypercalcemia (serum ionized Ca > 5.3 mg/dL), alkalosis (pH > 7.45), or acidosis (pH < 7.35).

Blood, tracheal, and urine cultures results were collected for the period from the initiation of CRRT to up to 28 days after CRRT initiation. In-hospital mortality was also evaluated.

### Outcomes

The main outcome of interest was to time to first filter clotting (in hours) during CRRT. Secondary outcomes included clotting at 24, 48, and 72 h, as well as (major and minor) bleeding episodes, the need for blood transfusion, and a drop in platelet count (to < 100,000/mm^3^), during the first filter use.

### Ethical aspects

The study was approved by the local institutional review board (Reference no. 33351120.0.0000.0068). This study was performed in accordance with the Strengthening the Reporting of Observational Studies in Epidemiology statement [13].

### Statistical analysis

Continuous variables are reported as mean ± SD or as median and interquartile range (IQR), as appropriate. Categorical variables are summarized as proportions. The COV − ACD-A only; COV + ACD-A only; and COV + ACD-A + UH groups were compared by analysis of variance or Kruskal–Wallis test, as appropriate, for continuous variables and by chi-square test or Fisher’s exact test for categorical variables. Differences were considered statistically significant at *p* < 0.05. Filter survival was analyzed with Kaplan–Meier estimates. The *p*-values were calculated by log-rank test.

Because there were only two patients in the COV − ACD-A + UH group, they were excluded from the analysis, given that such a small sample would have precluded any meaningful analysis and that the inclusion of such patients was not expected in the initial design of the study.

We plotted additional Kaplan–Meier curves in an exploratory analysis of different scenarios, including CVVH patients excluded in the primary analysis, and restricting the analysis to specific subgroups. We used Cox proportional hazards analysis to evaluate the association between each anticoagulation strategy and circuit clotting risk, adjusting for covariates. Hazard ratios (HRs) and the corresponding 95% confidence intervals (CIs) were calculated. Reported *p*-values in the Cox model are based on the Wald test. Models 1 and 2 included all 238 subjects and were built on the basis of variables of clinical relevance. Model 3 was restricted to the 180 patients with COVID-19-associated AKI in whom D-dimer levels were measured.

Statistical analyses were performed and graphics were generated with the R statistical software, version 4.0.2 (R Development Core Team, 2020).

## Results

**Fig. 1:**
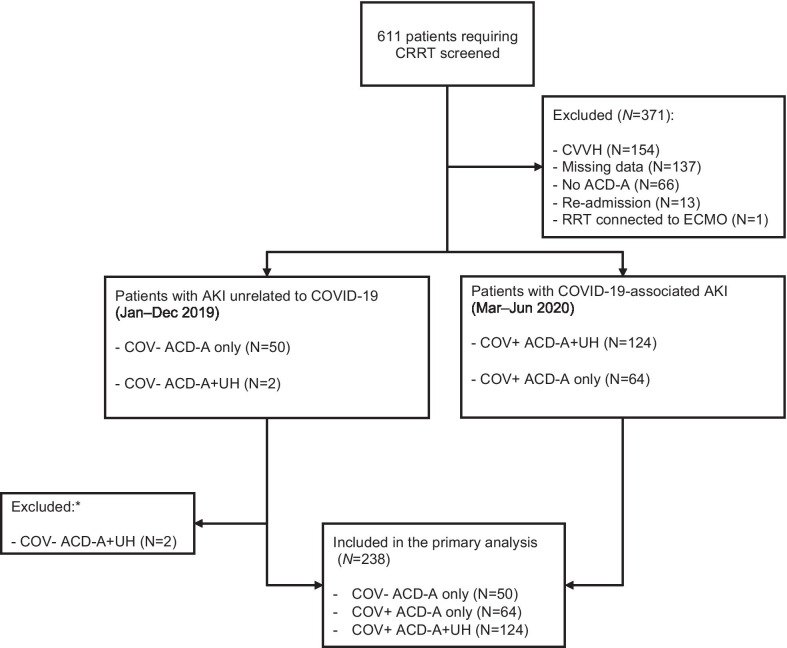
Flowchart of the study population selection process. CRRT, continuous renal replacement therapy; CVVH, continuous venovenous hemofiltration; ACD-A, anticoagulant citrate dextrose solution formula A; RRT, renal replacement therapy; ECMO, extracorporeal membrane oxygenation; AKI, acute kidney injury; COVID-19, coronavirus disease 2019; COV−, not diagnosed with coronavirus disease 2019; COV+, diagnosed with coronavirus disease 2019; UH, unfractionated heparin. *Because there were only 2 patients in this category, which precluded any meaningful analysis, and because the initial study design did not allow for the inclusion of such patients

We selected 238 patients. A flowchart of the patient selection process is shown in Fig. 1. Distribution of CRRT modalities regarding COVID-19 status and use of heparin are shown in Additional file 1: Table S1. Table 1 shows the baseline characteristics of the patients, by COVID-19 status and heparin use. In brief, the patients with COVID-19 were older; the prevalence of hypertension, diabetes, and obesity was also higher among those patients. In the COV + groups, the median arterial oxygen tension/fraction of inspired oxygen ratio was lower and vasopressor use was more common. In patients without intravenous heparin, COV + patients received more prophylactic subcutaneous heparin than COV − group.

**Table 1:** Characteristics of patients undergoing continuous renal replacement therapy, by coronavirus 2019 disease status and anticoagulation strategy

Characteristic	COV −	COV +		*p*
	ACD-A only	ACD-A only	ACD-A + UH	
	(*n* = 50)	(*n* = 64)	(*n* = 124)	
Age, years, median (IQR)	54.3 (42.1–62.9)	64.5 (53.1–70.7)	63.6 (54.3–70.8)	< 0.001
Male sex, %	68	83	70	0.118
White, %	62	63	68	0.620
BMI > 30 kg/m^2^, %	4	27	24	0.005
Hypertension, %	46	69	64	0.034
Diabetes, %	22	45	45	0.012
Mechanical ventilation* %	78	98	95	< 0.001
PaO_2_/FiO_2_ ratio*^,a^, median (IQR)	340 (254–408)	165 (110–252)	160 (105–195)	< 0.001
Vasopressor use,* %	76	92	93	0.004
Serum creatinine,* mg/dl, median (IQR)	4.57 (3.15–6.55)	5.12 (3.78–6.20)	5.11 (3.56–7.15)	0.644
Serum BUN,* mg/dl, median (IQR)	76.4 (56.5–102)	103 (78.0–130)	102 (73.5–121)	0.003
Total bilirubin,*^,b^ mg/dl, median (IQR)	0.73 (0.31–2.11)	0.48 (0.35–0.76)	0.49 (0.30–0.76)	0.111
Hemoglobin,* g/dl, median (IQR)	8.90 (8.10–10.7)	9.90 (8.00–12.6)	9.95 (8.38–11.7)	0.246
Leukocytes,* 10^3^/mm^3^, median (IQR)	15.5 (9.11–20.5)	15.1 (11.6–22.1)	18.8 (12.6; 25.9)	0.028
Platelets,* 10^3^/mm^3^, median (IQR)	194 (100–326)	262 (135–364)	304 (201–365)	0.006
D-dimer closest to CRRT initiation,^c^ ng/ml, median (IQR)	N/A	6636 (4244–14,020)	5554 (1833–13,968)	0.254
D-dimer closest to filter clotting event,^†,d^ ng/ml, median (IQR)	N/A	6350 (3971–14,230)	6488 (3122−19,392)	0.736
CRRT modality				0.700
CVVHD, %	84	89	88	
CVVHDF, %	16	11	12	
Main parameters in CRRT, mean ± SD				
Blood flow, ml/min	162 ± 21.6	151 ± 12.1	150 ± 10.4	< 0.001
Dialysate flow, ml/h	1954 ± 508	2358 ± 498	2309 ± 442	< 0.001
Replacement flow,^‡^ ml/h	1025 ± 410	1057 ± 351	880 ± 243	0.393
Filtration fraction,^‡^ %	10.6 ± 6.45	21.7 ± 9.60	17.5 ± 4.95	0.010
Catheter location				0.007
Right internal jugular vein, %	42	47	56	
Left internal jugular vein, %	22	6	4	
Femoral vein, %	36	47	40	
Non-tunneled catheter, %	98	100	98	0.661
Subcutaneous heparin for VTE prophylaxis, %	58	84	35	< 0.001
Target citrate concentration				< 0.001
2–3 mmol/L, %	96	17	6	
4–5 mmol/L, %	4	83	94	
Mean post-filter ionized Ca,^e^ mg/dL^§^, median (IQR)	1.36 (1.19–1.48)	1.06 (0.91–1.23)	1.06 (0.94–1.18)	< 0.001
Mean post-filter ionized Ca < 1.0,^e^ %	12	36	35	0.017
Mean post-filter ionized Ca < 1.4,^e^ %	61	91	96	< 0.001

COV− , not diagnosed with coronavirus disease 2019; COV+ , diagnosed with coronavirus disease 2019; ACD-A, anticoagulant citrate dextrose solution formula A; UH, unfractionated heparin; IQR, interquartile range; BMI, body mass index; PaO_2_/FiO_2_, arterial oxygen tension/fraction of inspired oxygen; BUN, blood urea nitrogen; CRRT, continuous renal replacement therapy; SD, standard deviation; CVVHD, continuous venovenous hemodialysis; CVVHDF, continuous venovenous hemodiafiltration; VTE, venous thromboembolism

*At start of CRRT; ^†^Only in patients who presented filter clotting; ^‡^Only in patients submitted to CVVHDF (*n* = 30); ^§^Mean of all measurements during CRRT

^a^*n* = 229; ^b^*n* = 191; ^c^*n* = 181; ^d^*n* = 85; ^e^*n* = 197

Patients with a diagnosis of COVID-19 started CRRT at higher serum blood urea nitrogen values, despite similar serum creatinine levels. In the COV + groups, CRRT was performed with lower blood flow and a higher dialysate flow. In addition, the COV + patients received higher citrate dose as regional anticoagulation. The post-filter iCa was lower in COV + patients than in COV − patients (Table 1). Despite the fact that the proportion of patients with a high target citrate concentration was lower in the COV + ACD-A group than in the COV + ACD-A + UH group (*p* = 0.022), the post-filter iCa was comparable between the two groups (*p* = 0.699).

### Heparin, filter life, and bleeding-related adverse events

**Fig. 2:**
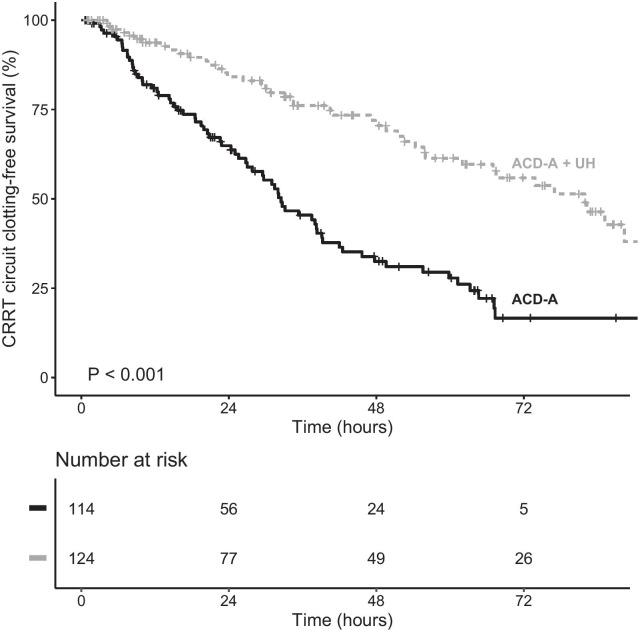
Kaplan–Meier estimate of filter clotting during the first filter use in continuous renal replacement therapy (CRRT) using anticoagulant citrate dextrose solution formula A (ACD-A), with and without unfractionated heparin (UH)

Of the patients in our sample, 71.8% received prefilter heparin and 28.2% received systemic heparin. Of the 238 patients evaluated, 111 (46.6%) experienced clotting-censored filter loss during the first filter use. Figure 2 depicts Kaplan–Meier estimates showing that filter survival was longer in patients receiving heparin than in the other patients.

**Fig. 3:**
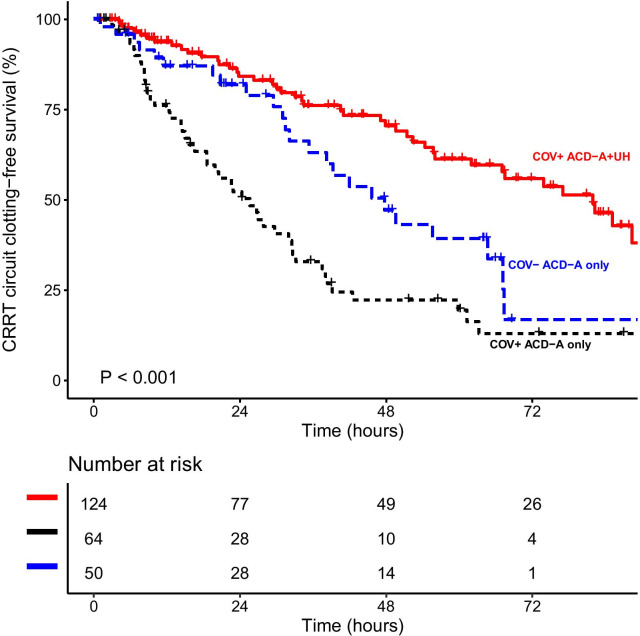
Kaplan–Meier estimate of filter clotting during the first filter use in continuous renal replacement therapy (CRRT) using anticoagulant citrate dextrose solution formula A (ACD-A), with and without unfractionated heparin (UH), by group

**Fig. 4:**
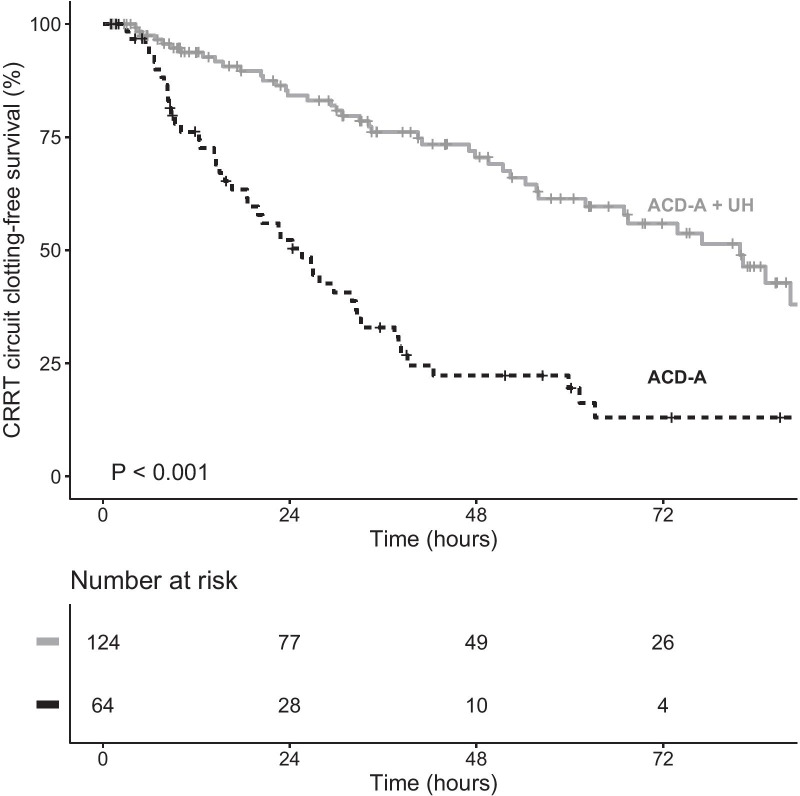
Kaplan–Meier estimate of filter clotting during the first filter use in continuous renal replacement therapy (CRRT) using anticoagulant citrate dextrose solution formula A (ACD-A), with and without unfractionated heparin (UH), among COVID-19-positive (COV +) patients (COV + ACD-A only group vs. COV + ACD-A + UH group)

Filter survival was longer in the COV + ACD-A + UH group than in the two other groups (Fig. 3). Likewise, when we analyzed only the patients diagnosed with COVID-19, we found that heparin still reduced the risk of filter clotting (Fig. 4). In comparison with ACD-A only, heparin was associated with a lower likelihood of filter clotting, whether it was given prefilter (HR = 0.45, 95% CI 0.24–0.86, *p* = 0.015) or systemically (HR = 0.35, 95% CI 0.23–0.54, *p* < 0.001), as shown in Additional file 1: Figure S1.

An exploratory analysis including the patients undergoing CVVH, who were excluded from the main analysis, showed that filter survival was still longer in the ACD-A + UH group than in the ACD-A only group (Additional file 1: Figure S2).

In our sample, filter survival was longer among the patients submitted to CVVHDF than among those submitted to CVVHD or CVVH (Additional file 1: Figure S3), and a comparison between CVVHD and CVVH suggested that CVVHD is better than CVVH regarding filter clotting (Additional file 1: Figure S4). However, because CVVHD was performed mainly in the COV + group, in which there was a high frequency of heparin use, and CVVH was performed mainly in the COV − group, in which the frequency of heparin use was low, we plotted a Kaplan–Meier curve restricted to COV − patients without the use of heparin. Additional file 1: Figure S5 shows that, within that subgroup, the clotting risk was similar for CVVH and CVVHD.

The median clotting-free filter survival was 47.7 h (lower limit of the 95% CI of 35.3) in the COV − ACD-A only group, 25.6 h (95% CI 18.6–33.2) in the COV + ACD-A only group, and 81.9 h (lower limit of the 95% CI of 62.0) in the COV + ACD-A + UH group. The 24-h clotting-free filter survival probability (95% CI) was 81.9% (71.2–94.2) in the COV − ACD-A only group, 52.2% (40.6–67.2) in the COV + ACD-A only group, and 84.2% (77.3–91.7) in the COV + ACD-A + UH group. The 48-h clotting-free filter survival probability (95% CI) was 47.1% (32.9–67.6) in the COV − ACD-A only group, 22.3% (13.3–37.3) in the COV + ACD-A only group, and 70.5% (61.6–80.8) in the COV + ACD-A + UH group. The 72-h clotting-free filter survival probability (95% CI) was 16.8% (5.5–51.4) in the COV − ACD-A only group, 13.0% (5.8–29.2) in the COV + ACD-A only group, and 55.9% (45.5–68.6) in the COV + ACD-A + UH group.

The rate of heparin-related adverse events was relatively low in our patient sample (Table 2). The difference among all groups was not significant regarding bleeding episodes (minor and major). A low platelet count and the need for blood transfusion were more common among the patients not diagnosed with COVID-19, although those parameters were similar between the COV + ACD-A + UH and COV + ACD-A only groups (Table 2). Although in-hospital mortality was higher in both COV + groups than in the COV − ACD-A only group (Table 2), it was comparable between the COV + ACD-A + UH and COV + ACD-A only groups.

**Table 2:** Adverse events in patients undergoing continuous renal replacement therapy, by anticoagulation strategy

Event	COV −	COV +		*p*	p*
	ACD-A only	ACD-A only	ACD-A + UH		
	(*n* = 50)	(*n* = 64)	(*n* = 124)		
Minor bleeding episode,^a^ %	8.0	1.7	4.2	0.319	0.665
Major bleeding episode,^a^ %	16.0	5.0	5.0	0.060	1.000
Blood transfusion, %	44.0	23.4	25.0	0.024	0.954
Platelet count < 100,000,^b^ %	42.0	20.3	13.4	< 0.001	0.335
Peak aPTT in the first 72 h of CRRT^c^, median (IQR)	1.14 (1.01–1.64)	1.30 (1.10–1.69)	2.42 (1.70–3.52)	< 0.001	< 0.001
In-hospital mortality, %	64.0	84.4	84.7	0.005	1.000
Hypokalemia,^d^ %	16.3	10.3	10.8	0.556	1.000
Hyperkalemia,^e^ %	16.0	6.4	12.4	0.269	0.482
Hypophosphatemia,^c^ %	20.5	13.2	19.8	0.548	0.742
Hyperphosphatemia,^f^ %	15.2	9.7	18.5	0.304	0.560
Hyponatremia,^g^ %	6.0	0.0	1.8	0.077	0.549
Hypernatremia,^h^ %	2.0	1.8	0.9	0.791	1.000
Hypocalcemia,^i^ %	28.3	29.0	22.5	0.559	0.849
Hypercalcemia,^j^ %	25.5	20.3	12.3	0.099	0.356
Alkalosis,^k^ %	35.4	17.0	17.7	0.028	1.000
Acidosis,^e^ %	14.3	12.9	13.9	0.974	1.000

COV−, not diagnosed with coronavirus disease 2019; COV+, diagnosed with coronavirus disease 2019; ACD-A, anticoagulant citrate dextrose solution formula A; UH, unfractionated heparin; aPTT, activated partial thromboplastin time, IQR, interquartile range; CRRT, continuous renal replacement therapy

^a^*n* = 229; ^b^*n* = 221; ^c^*n* = 193; ^d^*n* = 218, ^e^*n* = 233, ^f^*n* = 216, ^g^*n* = 215, ^h^*n* = 219, ^i^*n* = 228, ^j^*n* = 220, ^k^*n* = 214

*Kruskal–Wallis for COV + ACD-A only vs. COV + ACD-A + UH

It is noteworthy that neither the D-dimer levels collected closest to CRRT initiation nor those collected closest to the clotting event differed between the two COV + groups (Table 1).

### Other potential contributors to filter clotting

We analyzed D-dimer levels determined at two different time points: closest to CRRT initiation and closest to the clotting event. The median time between D-dimer determination closest to CRRT initiation and the actual initiation of CRRT was 47.9 (14.6–184.0) h, and there was no significant difference between the COV + ACD-A only group and the COV + ACD-A + UH group—47.9 (13.1–189.1) h and 45.3 (14.6–172.8) h, respectively. The D-dimer level was determined more often before CRRT initiation than after (Additional file 1: Table S2).

**Fig. 5:**
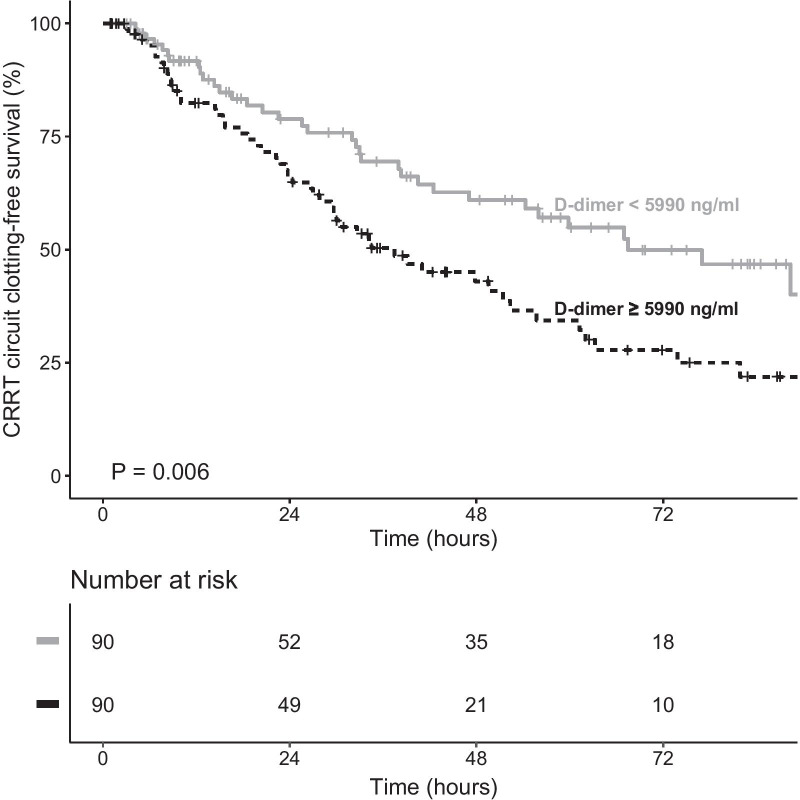
Kaplan–Meier estimate of filter clotting in the first filter use during continuous renal replacement therapy (CRRT), according to the D-Dimer median split

The results of the univariate Cox proportional hazards analysis are shown in Table 3. In the multivariate Cox models (Table 4), the risk of filter clotting was shown to be lower when heparin was used, even after adjustment for other covariates. Higher hemoglobin levels were associated with a higher risk of circuit clotting, as were high D-dimer levels. A D-dimer level above the median (5990 ng/ml) was associated with more than 2 times higher risk of filter clotting (Table 4). Figure 5 shows the Kaplan–Meier estimates for filter survival, by D-dimer level.

**Table 3:** Cox univariate proportional-hazards analysis for filter clotting during first filter use

Risk factor	All patients	*p*
	(*N* = 238)	
	HR (95% CI)	
Heparin use	0.37 (0.25–0.55)	< 0.001
COVID-19 diagnosis	0.87 (0.55–1.38)	0.561
Age, per year increase	1.01 (1.00–1.03)	0.144
Male sex	1.28 (0.83–1.96)	0.266
D-dimer level,* per 1000 increase	1.01 (1.00–1.02)	0.003
Hemoglobin level	1.12 (1.04–1.21)	0.003
Platelet count, per 100,000 increase	1.00 (1.00–1.00)	0.299
BUN, per 10 increase	1.00 (0.95–1.04)	0.922
ACD-A 4–5 mmol/L vs. 2–3 mmol/L	0.82 (0.54–1.23)	0.331
Vasopressor use	0.93 (0.51–1.70)	0.826
Treatment modality	0.55 (0.27–1.14)	0.109
Obesity	1.09 (0.70–1.69)	0.703

HR, hazard ratio; COVID-19, coronavirus disease 2019, BUN, blood urea nitrogen; ACD-A, anticoagulant citrate dextrose solution formula A

*Value obtained closest to the initiation of renal replacement therapy (median 5990 ng/ml)

**Table 4:** Multivariate Cox regression models for first continuous renal replacement therapy circuit clotting in 72 h

Factor	Model 1		Model 2*		Model 3^†^	
	HR (95% CI)	*p*	HR (95% CI)	*p*	HR (95% CI)	*p*
Anticoagulation (ACD-A + UH vs. ACD-A only)	0.28 (0.18–0.43)	< 0.001	0.25 (0.16–0.39)	< 0.001	0.28 (0.18–0.43)	< 0.001
Adjustment covariate						
COVID-19 diagnosis	2.02 (0.96–4.24)	0.063	2.15 (0.99–4.68)	0.053		
ACD-A dose (4–5 mmol/L vs. 2–3 mmol/L)	0.88 (0.46–1.69)	0.699	0.72 (0.36–1.42)	0.344		
Hemoglobin level (per 1 g/dl increase)	1.13 (1.05–1.22)	0.001	1.18 (1.08–1.28)	< 0.001		
Platelet count (per 100,000 increase)	1.00 (1.00–1.00)	0.015	1.00 (1.00–1.00)	0.070		
Age (per year increase)			1.01 (1.00–1.03)	0.115		
Male sex			0.91 (0.57–1.44)	0.677		
BUN (per unit increase)			1.00 (0.99–1.00)	0.379		
ACD-A dose (4 mmol/L vs. 3 mmol/L)			0.72 (0.36–1.42)	0.344		
Median D-dimer level^‡^ (≥ 5990 ng/ml vs. < 5990 ng/ml)					1.94 (1.25–3.01)	0.003

HR, hazard ratio; ACD-A, anticoagulant citrate dextrose solution formula A; UH, unfractionated heparin; COVID-19, coronavirus disease 2019; BUN, blood urea nitrogen

*Also adjusted for CRRT modality strata; ^†^analysis restricted to the 180 patients for whom D-dimer values were available; ^‡^value obtained closest to the initiation of renal replacement therapy (median 5990 ng/ml)

In the multivariable Cox regression analysis, neither the ACD-A dose level nor the CRRT modality represented risk factors for filter clotting (Table 4). A diagnosis of COVID-19 was associated with a nonsignificantly higher risk of filter clotting (HR = 2.15, 95% CI 0.99–4.68, *p* = 0.053).

### Other CRRT-related adverse effects

The main electrolyte disturbances are shown in Table 2. The median electrolyte levels during CRRT are shown in Additional file 1: Table S3.

There were few culture-proven infections in our patient sample, and there were no differences between groups regarding blood or urine cultures. Positive tracheal cultures were more common in the COV + groups than in the COV − ACD-A only group, although there were no differences regarding heparin use (Additional file 1: Table S4). Time to filter clotting might have had a small impact on infection rates in our sample. The time from the initiation of CRRT to filter clotting was found to correlate weakly with the number of positive cultures within the first 28 days after CRRT initiation (*r* = 0.13, *p* = 0.043), as shown in Additional file 1: Figure S6. The main reasons for a filter change are shown in Additional file 1: Table S5.

### Discussion

The key finding of this study is that RCA plus infusion of unfractionated heparin might be superior to RCA alone for prolonging circuit life and reducing filter losses during CRRT in COV + AKI patients, with similar rates of adverse events (bleeding or the need for blood transfusion). In addition to the increased demand for dialysis, COVID-19 patients are especially predisposed to thrombotic events, theoretically increasing the propensity for filter clotting [9, 14–16]. CRRT filter clotting is a major concern in critically ill patients because it not only can result in shortages of medical equipment and consumables but also may be associated with blood loss and shorter dialysis times. Therefore, it is crucial to maintain appropriate, effective anticoagulation during dialysis [(17, 18].

Although it was not the main focus of this study, the exploratory analysis restricted to the subgroup of heparin-free patients without COVID-19 and including CVVH patients showed that CVVH did not differ from CVVHD regarding filter clotting, contrary to our expectations. There could be a number of explanations for that. Perhaps the clotting risk is in fact similar between CVVH and CVVHD. It is also possible that the comparison was underpowered to detect a difference between these modalities because of the small number of patients in the subgroup. In addition, the apparent lack of a difference might be the consequence of confounders such as the use of different dialysis machines to perform CVVH, CVVHD, and CVVHDF, which is a common practice at our facility.

Various anticoagulation strategies have been studied [19]. Randomized controlled trials have shown RCA to be clearly superior to the use of heparin, with a better adverse-event profile [19–22]. Unless contraindicated, citrate is also recommended as the first-line option in CRRT [12]. However, none of those studies involved COV + AKI patients or other known prothrombotic factors. In addition, there have been no studies comparing the use of the combination of citrate and heparin with the use of either of those anticoagulation strategies, even in patients without COVID-19.

Our findings suggest that the use of systemic heparin plus RCA blunts the excessive prothrombotic effect that RRT has on filter patency in COVID-19 patients [9, 15, 16]. Although some groups are already using this strategy informally in the management of CRRT in COVID-19 patients [9, 10, 14, 16], there have been few studies comparing different anticoagulation strategies in that context.

Our data are in agreement with the findings of Shankaranarayanan et al. [10], who showed that, in COVID-19 patients, the concomitant use of systemic heparin and citrate could lead to fewer thrombotic events in CRRT circuits when compared with other strategies, including citrate alone and heparin alone. Those authors reported a median filter life of just 21 h for no anticoagulation, compared with 40 h for citrate alone and > 72 h for citrate plus heparin. Similarly, in the present study, in which no procedures were performed without anticoagulation, the median filter survival was 25.6 h for citrate alone and 81.9 h for heparin associated with citrate. Wen et al. [16] also showed longer circuit life when heparin-based regimens were used in sustained low-efficiency dialysis.

In the present study, the adverse-event profile was similar between the ACD-A only and ACD-A + UH groups. Heparin use was not found to be associated with lower platelet counts. We found a low incidence of bleeding episodes, so it is possible that they may not be sufficient to determine if adverse events rates are certainly different between groups. In addition, our center usually treats a high number of patients with liver diseases, although that was not the case during the COVID-19 pandemic of 2020. Therefore, despite using more heparin, we had fewer patients with comorbidities related to coagulopathy in the COV + groups, which could explain why platelet counts were lower and more blood transfusions were required in the COV − group patients. However, despite the relatively small patient sample, ours is the largest study conducted to date regarding anticoagulation in CRRT in patients with COVID-19. To our knowledge, this is also the first report of the safety profile of the heparin-citrate combination in CRRT. We also found that in-hospital mortality was similar to that previously reported for patients with AKI, with or without a diagnosis of COVID-19 [23, 24], COVID-19-associated AKI being the more lethal of the two.

Another important aspect of the thrombotic potential of COVID-19 is related to D-dimer levels [1]. In COVID-19 patients, an elevated D-dimer level has been shown to be predictive of thrombotic complications [25]. In the present study, elevated D-dimer levels were found to predispose to higher rates of filter clotting in CRRT. That differs from the findings of a previous study comparing diverse anticoagulation strategies in COVID-19 [16], in which D-dimer levels had no apparent effect on circuit clotting. However, that study evaluated only sustained low-efficiency dialysis, with a median of < 36 h per session in all groups, and the reported D-dimer levels were much lower. Therefore, it was not possible to draw comparisons with the present study.

To our knowledge, ours is the largest study comparing ACD-A alone and ACD-A plus unfractionated heparin in CRRT performed in COVID-19 patients. We believe that it is also the first study to address safety concerns regarding the use of the latter combination.

Our study has some limitations. To meet the challenge of the potential for clotting in CRRT performed in COVID-19 patients, we not only added systemic heparin to the regimen but also increased the ACD-A concentration and lowered the target post-filter iCa concentration. Therefore, in the COV + ACD-A + UH group, the proportion of patients in whom the target citrate concentration was 4–5 mmol/L (rather than 2–3 mmol/L) was higher. That outcome may in part be a consequence of the success of the anticoagulation with ACD-A, which could reduce its generalizability. That could have influenced our results, although a target citrate concentration of 4–5 mmol/L was not found to be a protective factor in the multivariate Cox analysis and did not lead to lower post-filter iCa when compared with that observed for the COV + ACD-A only group. However, the addition of heparin was found to be consistently associated with a lower risk of filter clotting, and we believe that this combined strategy should be adopted in settings in which there is a high risk of clotting. In addition, because of the retrospective study design, data regarding D-dimer levels were not available for the COV − AKI patients. It would have been interesting to determine whether higher D-dimer levels are associated with higher mortality. That would also have allowed us to investigate whether the higher risk of filter clotting in COV + AKI patients is attributable solely to D-dimer levels. Furthermore, also because of the retrospective study design, our results might be attributable to other, unmeasured co-interventions. Moreover, our study reflects the experience of a single center in Brazil and therefore may not reflect the reality for all COV + AKI patients.

## Conclusions

In conclusion, the combination of systemic heparin and RCA appears to extend filter life in COV + AKI patients. We hypothesize that this strategy would be useful in any patients who are prone to coagulation events. Prospective trials are needed in order to confirm or refute our findings.

## Supplementary Information

**Additional file 1. Table S1** Distribution of continuous renal replacement therapy modalities, by coronavirus disease 2019 status and heparin use. **Table S2** Timing of collection of D-dimer level determination closest to the initiation of continuous renal replacement therapy, restricted to the patients diagnosed with coronavirus disease 2019 for whom D-dimer values were available (n=180). **Table S3** Electrolyte profiles during continuous renal replacement therapy. **Table S4** Mean numbers of positive cultures within the first 28 days after the initiation of continuous renal replacement therapy. **Table S5** Main reasons for a filter change. **Figure S1** Kaplan–Meier estimate of filter clotting during the first filter use in continuous renal replacement therapy (CRRT) using anticoagulant citrate dextrose solution formula A (ACD-A), with and without unfractionated heparin, the former subdivided by the type of heparin use. **Figure S2** Kaplan–Meier estimate of filter clotting during the first filter use in continuous renal replacement therapy (CRRT) using anticoagulant citrate dextrose solution formula A (ACD-A), with and without unfractionated heparin (UH) in the first filter use during continuous renal replacement therapy (CRRT), including the 154 patients who were excluded for undergoing continuous venovenous hemofiltration. **Figure S3** Kaplan–Meier estimate of filter clotting during continuous renal replacement therapy (CRRT), by modality, including the patients who were excluded for undergoing continuous venovenous hemofiltration (CVVH). **Figure S4** Kaplan–Meier estimate of filter clotting during continuous renal replacement therapy (CRRT), comparing continuous venovenous hemodialysis (CVVHD) with continuous venovenous hemofiltration (CVVH). **Figure S5** Kaplan–Meier estimate of filter clotting during heparin-free continuous renal replacement therapy (CRRT) in patients without coronavirus disease 2019, comparing continuous venovenous hemodialysis (CVVHD) with continuous venovenous hemofiltration (CVVH). **Figure S6** Correlation between the time from the initiation of continuous renal replacement therapy (CRRT) to filter clotting and any of the infections studied.

## Data Availability

The datasets used and/or analyzed during the current study are available from the corresponding author on reasonable request.

## Abbreviations

ACD-A: Anticoagulant citrate dextrose solution formula A
AKI: Acute kidney injury
aPTT: Activated partial thromboplastin time
CI: Confidence interval
COVID-19: Coronavirus disease 2019
COV + AKI: Acute kidney injury associated with coronavirus disease 2019
COV − AKI: Acute kidney injury not associated with coronavirus disease 2019
CRRT: Continuous renal replacement therapy
CVVH: Continuous venovenous hemofiltration
CVVHD: Continuous venovenous hemodialysis
CVVHDF: Continuous venovenous hemodiafiltration
HR: Hazard ratio
iCa: Ionized calcium
ICU: Intensive care unit
KDIGO: Kidney Disease Improving Global Outcomes
RCA: Regional citrate anticoagulation
RRT: Renal replacement therapy
SARS-CoV-2: Severe acute respiratory syndrome coronavirus 2
SD: Standard deviation
UH: Unfractioned heparin
