# Supplementary material for: Continuous renal replacement therapy in COVID-19—associated AKI: adding heparin to citrate to extend filter life—a retrospective cohort study
Source: Crit Care. 2021 Aug 19;25:299. doi: 10.1186/s13054-021-03729-9 (PMC8375288; doi:10.1186/s13054-021-03729-9)
Supplement: Supplementary file 1 — Additional file 1. Table S1 Distribution of continuous renal replacement therapy modalities, by coronavirus disease 2019 status and heparin use. Table S2 Timing of collection of D-dimer level determination closest to the initiation of continuous renal replacement therapy, restricted to the patients diagnosed with coronavirus disease 2019 for whom D-dimer values were available (n=180). Table S3 Electrolyte profiles during continuous renal replacement therapy. Table S4 Mean numbers of positive cultures within the first 28 days after the initiation of continuous renal replacement therapy. Table S5 Main reasons for a filter change. Figure S1 Kaplan–Meier estimate of filter clotting during the first filter use in continuous renal replacement therapy (CRRT) using anticoagulant citrate dextrose solution formula A (ACD-A), with and without unfractionated heparin, the former subdivided by the type of heparin use. Figure S2 Kaplan–Meier estimate of filter clotting during the first filter use in continuous renal replacement therapy (CRRT) using anticoagulant citrate dextrose solution formula A (ACD-A), with and without unfractionated heparin (UH) in the first filter use during continuous renal replacement therapy (CRRT), including the 154 patients who were excluded for undergoing continuous venovenous hemofiltration. Figure S3 Kaplan–Meier estimate of filter clotting during continuous renal replacement therapy (CRRT), by modality, including the patients who were excluded for undergoing continuous venovenous hemofiltration (CVVH). Figure S4 Kaplan–Meier estimate of filter clotting during continuous renal replacement therapy (CRRT), comparing continuous venovenous hemodialysis (CVVHD) with continuous venovenous hemofiltration (CVVH). Figure S5 Kaplan–Meier estimate of filter clotting during heparin-free continuous renal replacement therapy (CRRT) in patients without coronavirus disease 2019, comparing continuous venovenous hemodialysis (CVVHD) with continuous venovenous hemofilt [file 13054_2021_3729_MOESM1_ESM.docx]

**Additional file 1**

**Table S1** Distribution of continuous renal replacement therapy modalities, by coronavirus disease 2019 status and heparin use

| Modality | All patients | COV− | | COV+ | |
| --- | --- | --- | --- | --- | --- |
|  |  | ACD-A only | ACD-A+UH | ACD-A only | ACD-A+UH |
|  | (*N*=394)* | (*n*=194) | (*n*=9) | (*n*=66) | (*n*=125) |
| CVVHD, *n* (%) | 210 (53.3) | 42 (21.6) | 2 (22.2) | 57 (86.4) | 109 (87.2) |
| CVVHDF, *n* (%) | 30 (7.6) | 8 (4.1) | 0 (0) | 7 (10.6) | 15 (12.0) |
| CVVH, *n* (%) | 154 (39.1) | 144 (74.2) | 7 (77.8) | 2 (3.0) | 1 (0.8) |

COV−, not diagnosed with coronavirus disease 2019; COV+, diagnosed with coronavirus disease 2019; ACD-A, anticoagulant citrate dextrose solution formula A; UH, unfractionated heparin; CVVHD, continuous venovenous hemodialysis; CVVHDF, continuous venovenous hemodiafiltration; CVVH, continuous venovenous hemofiltration.

**Ex post facto* exclusions: no ACD-A (*n*=66), readmission (*n*=13), and renal replacement therapy connected to an extracorporeal membrane oxygenation circuit (*n*=1).

**Table S2** Timing of collection of D-dimer level determination closest to the initiation of continuous renal replacement therapy, restricted to the patients diagnosed with coronavirus disease 2019 for whom D-dimer values were available (*n*=180)

|  | COV+ ACD-A | | COV+ ACD-A+UH | |
| --- | --- | --- | --- | --- |
|  | (*n*=63) | | (*n*=117) | |
|  | Pre-CRRT | Post-CRRT | Pre-CRRT | Post-CRRT |
|  | *n*=51 (81%) | *n* =12 (19%) | *n*=93 (79%) | *n*=24 (21%) |
| Time (h) between D-dimer determination and CRRT initiation, median (IQR) | 60.4 (18.2–214) | 20.6 (2.0–46.2) | 79.8 (23.4–219) | 13.8 (2.1–28.9) |

COV+, diagnosed with coronavirus disease 2019; ACD-A, anticoagulant citrate dextrose solution formula A; UH, unfractionated heparin; CRRT, continuous renal replacement therapy; IQR, interquartile range.

**Table S3** Electrolyte profiles during continuous renal replacement therapy

| Variable | COV− | COV+ | | *p* |
| --- | --- | --- | --- | --- |
|  | ACD-A only | ACD-A only | ACD-A+UH |  |
|  | (*n*=50) | (*n*=64) | (*n*=124) |  |
| Sodium (mEq/L),* median (IQR) | 139 (136–143) | 138 (136–141) | 137 (134–140) | 0.061 |
| Potassium (mEq/L),^†^ median (IQR) | 4.38 (4.01–4.80) | 4.53 (4.25–5.02) | 4.53 (4.16–5.07) | 0.100 |
| Phosphorus (mg/dL),^‡^ median (IQR) | 4.07 (3.00–5.01) | 4.25 (3.60–5.10) | 4.43 (3.48–5.52) | 0.241 |
| pH,^§^ median (IQR) | 7.40 (7.36–7.42) | 7.30 (7.25–7.36) | 7.30 (7.22–7.36) | <0.001 |
| Ionized calcium (mg/dL),^║^ median (IQR) | 4.62 (4.37–4.97) | 4.60 (4.39–4.91) | 4.54 (4.23–4.84) | 0.239 |

COV−, not diagnosed with coronavirus disease 2019; COV+, diagnosed with coronavirus disease 2019; ACD-A, anticoagulant citrate dextrose solution formula A; UH, unfractionated heparin; IQR, interquartile range.

**n*=214; ^†^*n*=217; ^‡^*n*=191; ^§^*n*=214; ^║^*n*=218.

**Table S4** Mean numbers of positive cultures within the first 28 days after the initiation of continuous renal replacement therapy

| Positive culture | COV− | COV+ | | *p* |
| --- | --- | --- | --- | --- |
|  | ACD-A only | ACD-A only | ACD-A+UH |  |
|  | (*n*=50) | (*n*=64) | (*n*=124) |  |
| Blood,* mean ± SD | 0.52 ± 0.74 | 0.63 ± 0.90 | 0.40 ± 0.74 | 0.158 |
| Tracheal,^†^ mean ± SD | 0.13 ± 0.34 | 0.44 ± 0.59 | 0.31 ± 0.70 | 0.048 |
| Urine,^‡^ mean ± SD | 0.32 ± 0.59 | 0.22 ± 0.58 | 0.22 ± 0.49 | 0.497 |

COV−, not diagnosed with coronavirus disease 2019; COV+, diagnosed with coronavirus disease 2019; ACD-A, anticoagulant citrate dextrose solution formula A; UH, unfractionated heparin; SD, standard deviation.

**n*=237; ^†^*n*=212; ^‡^*n*=237.

**Table S5** Main reasons for a filter change*

| Reason | COV− | COV+ | |
| --- | --- | --- | --- |
|  | ACD-A only | ACD-A only | ACD-A+UH |
|  | *n*=50 | *n*=64 | *n*=124 |
| Clotting, % | 48.0 | 70.3 | 33.9 |
| CRRT device problems, % | 0.0 | 0.0 | 0.8 |
| Routine, % | 2.0 | 0.0 | 4.8 |
| Hemodynamic instability/intolerance, % | 14.0 | 15.6 | 35.5 |
| Physician decision to interrupt CRRT, % | 16.0 | 9.4 | 19.4 |
| Patient transfer, % | 14.0 | 0.0 | 2.4 |
| Vascular access problems, % | 6.0 | 4.7 | 3.2 |

**p* < 0.05 overall.

*

*

**Fig. S1** Kaplan–Meier estimate of filter clotting during the first filter use in continuous renal replacement therapy (CRRT) using anticoagulant citrate dextrose solution formula A (ACD-A), with and without unfractionated heparin, the former subdivided by the type of heparin use.


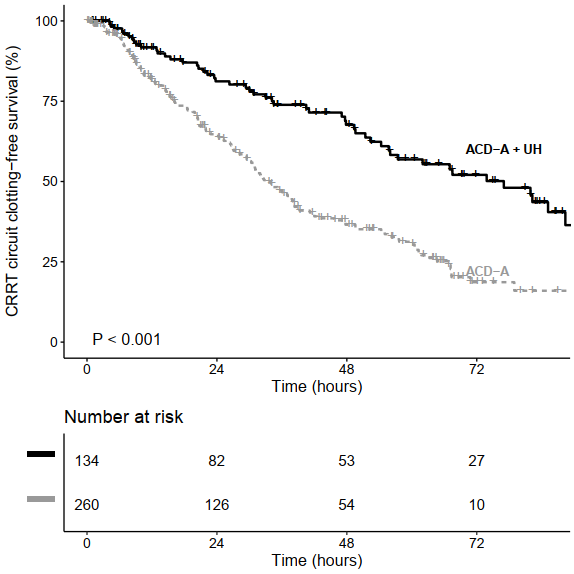


**Fig. S2** Kaplan–Meier estimate of filter clotting during the first filter use in continuous renal replacement therapy (CRRT) using anticoagulant citrate dextrose solution formula A (ACD-A), with and without unfractionated heparin (UH) in the first filter use during continuous renal replacement therapy (CRRT), including the 154 patients who were excluded for undergoing continuous venovenous hemofiltration.


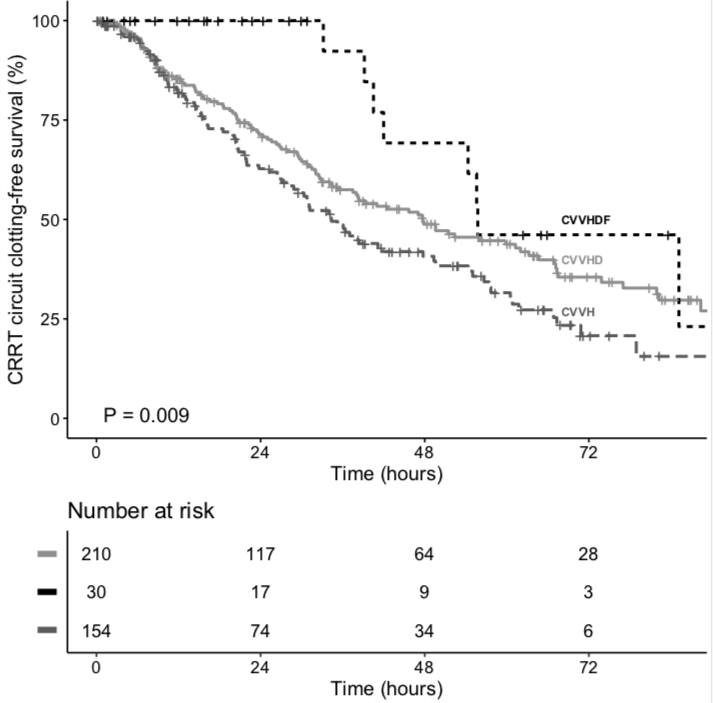


**Fig. S3** Kaplan–Meier estimate of filter clotting during continuous renal replacement therapy (CRRT),* by modality, including the patients who were excluded for undergoing continuous venovenous hemofiltration (CVVH).

CVVHD, continuous venovenous hemodialysis; CVVHDF, continuous venovenous hemodiafiltration.

*Exclusions: no ACD-A (*n*=66), readmission (*n*=13), and renal replacement therapy connected to an extracorporeal membrane oxygenation circuit (*n*=1).

**
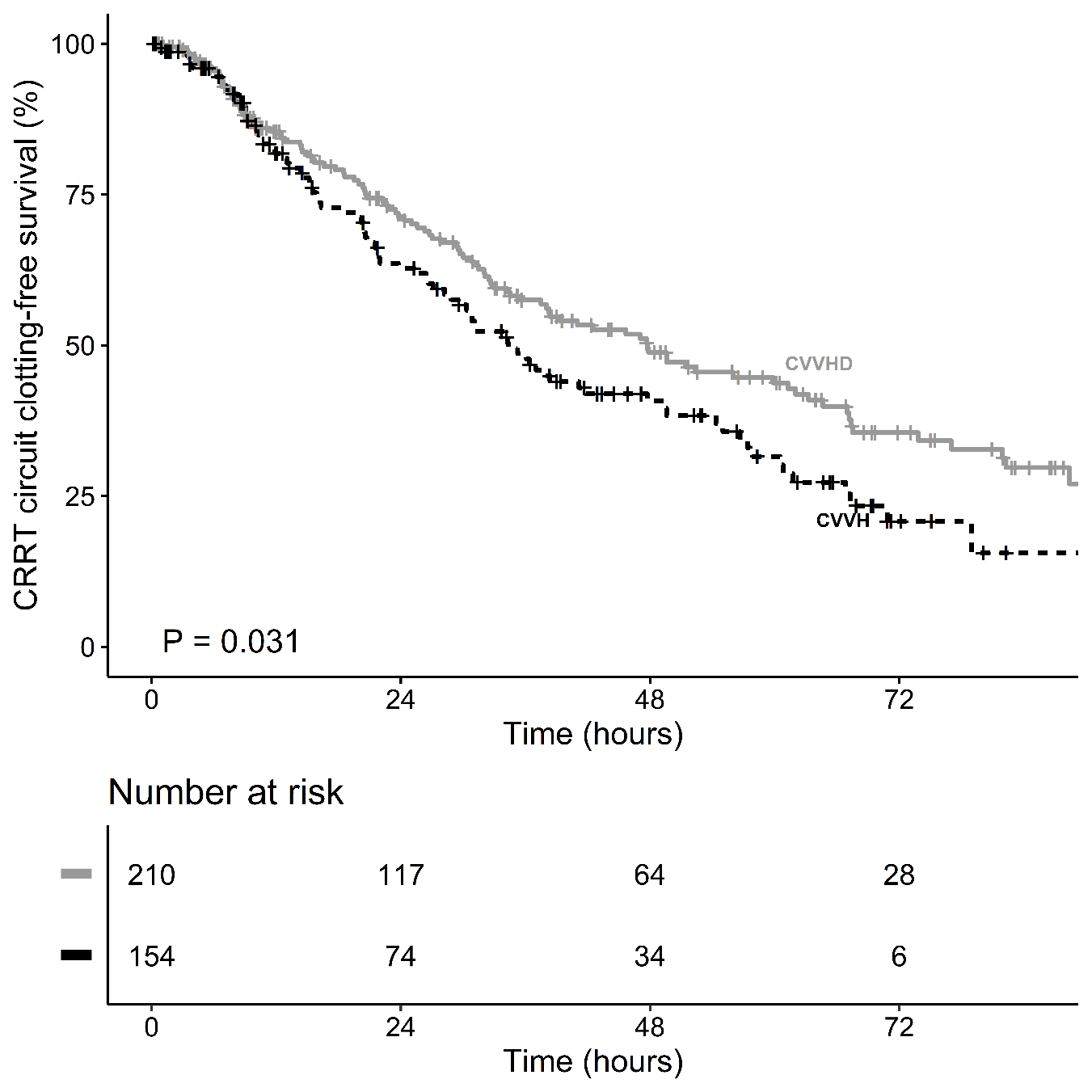
**

**Fig. S4** Kaplan–Meier estimate of filter clotting during continuous renal replacement therapy (CRRT), comparing continuous venovenous hemodialysis (CVVHD) with continuous venovenous hemofiltration (CVVH).

*Exclusions: no ACD-A (*n*=66), readmission (*n*=13), renal replacement therapy connected to an extracorporeal membrane oxygenation circuit (*n*=1); and continuous venovenous hemodiafiltration (*n*=30).


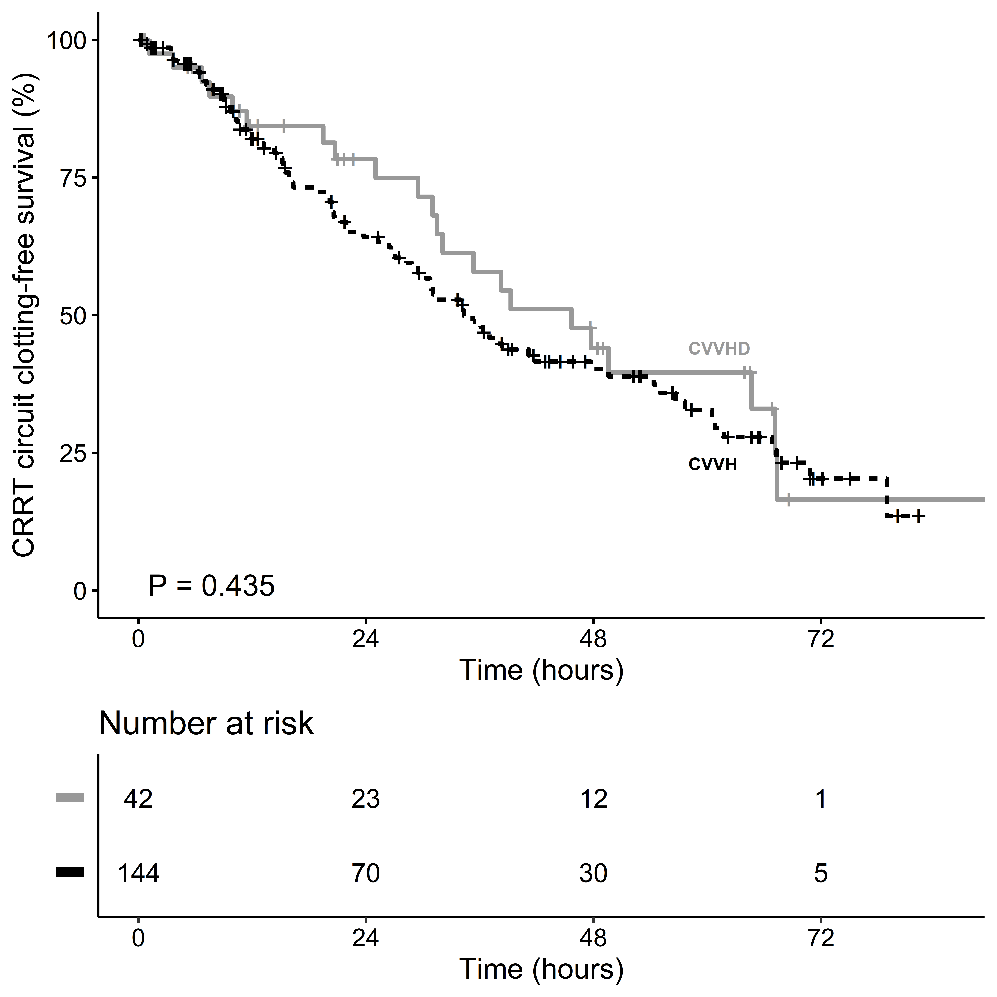


**Fig. S5** Kaplan–Meier estimate of filter clotting during heparin-free continuous renal replacement therapy (CRRT) in patients without coronavirus disease 2019, comparing continuous venovenous hemodialysis (CVVHD) with continuous venovenous hemofiltration (CVVH).

*Exclusions: no ACD-A (*n*=66), readmission (*n*=13), renal replacement therapy connected to an extracorporeal membrane oxygenation circuit (*n*=1); continuous venovenous hemodiafiltration (*n*=30); and a diagnosis of coronavirus disease 2019 or heparin use in patients without coronavirus disease 2019 (*n*=178).


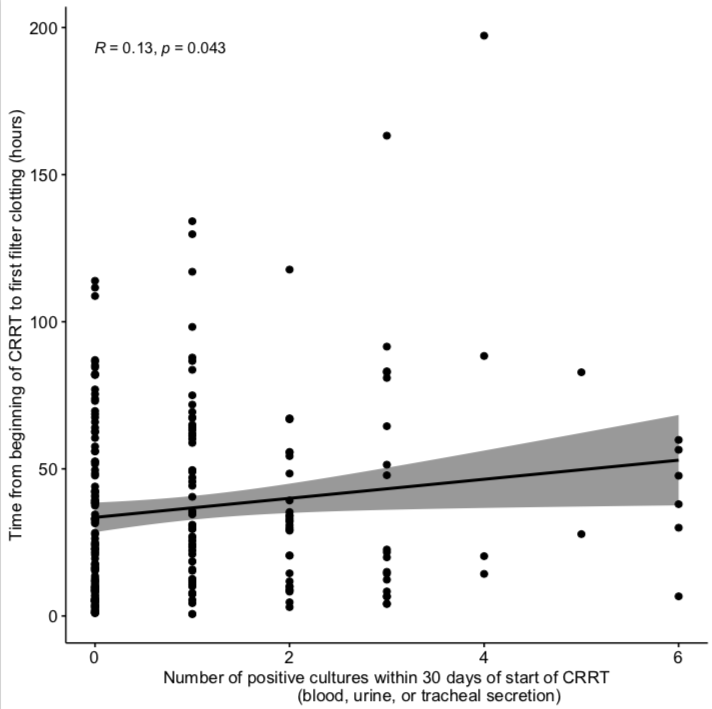


**Fig. S6** Correlation between the time from the initiation of continuous renal replacement therapy (CRRT) to filter clotting and any of the infections studied.
